# Alteration of TEAD1 Expression Levels Confers Apoptotic Resistance through the Transcriptional Up-Regulation of Livin

**DOI:** 10.1371/journal.pone.0045498

**Published:** 2012-09-24

**Authors:** André Landin Malt, Julie Cagliero, Kevin Legent, Joël Silber, Alain Zider, Domenico Flagiello

**Affiliations:** Univ Paris Diderot, Sorbonne Paris Cité, Equipe de Génétique Moléculaire de la Différenciation, IJM, UMR 7592 CNRS, Paris, France; Institute of Molecular and Cell Biology, Singapore

## Abstract

**Background:**

TEA domain (TEAD) proteins are highly conserved transcription factors involved in embryonic development and differentiation of various tissues. More recently, emerging evidences for a contribution of these proteins towards apoptosis and cell proliferation regulation have also been proposed. These effects appear to be mediated by the interaction between TEAD and its co-activator Yes-Associated Protein (YAP), the downstream effector of the Hippo tumour suppressor pathway.

**Methodology/Principal Findings:**

We further investigated the mechanisms underlying TEAD-mediated apoptosis regulation and showed that overexpression or RNAi-mediated silencing of the TEAD1 protein is sufficient to protect mammalian cell lines from induced apoptosis, suggesting a proapoptotic function for TEAD1 and a non physiological cytoprotective effect for overexpressed TEAD1. Moreover we show that the apoptotic resistance conferred by altered TEAD1 expression is mediated by the transcriptional up-regulation of Livin, a member of the Inhibitor of Apoptosis Protein (IAP) family. In addition, we show that overexpression of a repressive form of TEAD1 can induce Livin up-regulation, indicating that the effect of TEAD1 on Livin expression is indirect and favoring a model in which TEAD1 activates a repressor of Livin by interacting with a limiting cofactor that gets titrated upon TEAD1 up-regulation. Interestingly, we show that overexpression of a mutated form of TEAD1 (Y421H) implicated in Sveinsson's chorioretinal atrophy that strongly reduces its interaction with YAP as well as its activation, can induce Livin expression and protect cells from induced apoptosis, suggesting that YAP is not the cofactor involved in this process.

**Conclusions/Significance:**

Taken together our data reveal a new, Livin-dependent, apoptotic role for TEAD1 in mammals and provide mechanistic insight downstream of TEAD1 deregulation in cancers.

## Introduction

TEAD1 belongs to the family of conserved eukaryotic transcription factors (TEAD proteins), characterized by the TEA/ATTS DNA binding domain [1], [2], [3]. There are four closely related *Tead* genes (*Tead1* to *Tead4*) in mammals [4], [5] and one, *scalloped* (*sd*), in *Drosophila* [6]. The transcriptional activity of TEAD proteins requires their interaction with transcriptional co-activators [7], [8], [9]. In *Drosophila*, recent studies have demonstrated that Sd interacts with Yorkie (Yki) [10], [11], [12]. Yki is the *Drosophila* ortholog of mammalian YAP (Yes-Associated Protein) which *in vitro* and *in vivo* is a well characterized cofactor of the mammalian TEAD proteins [13], [14], [15], [16]. Both Yki and YAP, are the effectors of the Hippo tumour suppressor pathway that restricts organ growth by keeping in check cell proliferation and promoting apoptosis in *Drosophila* and in mammals [17], [18]. The regulation of Yki/YAP activity is achieved through direct phosphorylation by the Warts/Large Tumour Suppressor (LATS) kinases that are activated by the upstream components of the Hippo pathway and subsequently induce Yki/YAP cytoplasmic retention and inactivation [19], [20], [21]. Conversely,Yki overexpression promotes organ growth by stimulating cell proliferation and preventing apoptosis [19], [21]. This is achieved in *Drosophila* through the transcriptional induction of target genes including *Cyclin E*, *dE2F1* [10], the *bantam* microRNA [22], [23], *dmyc* [24], [25] and the *Drosophila inhibitor of apoptosis protein 1* (*Diap1*) [17]. However, although the framework of the Hippo signaling cascade is conserved between *Drosophila* and mammals there is still significant ambiguity as to how the pathway converges onto transcriptional regulators and elicits coherent transcriptional outcomes. For example, although both Yki and YAP promote cell and tissue growth in *Drosophila* and mammals, by interacting with the TEAD proteins, their target genes are not identical. For instance *Cyclin E* is induced by Yki overexpression in *Drosophila* [19], but not by YAP overexpression in mammalian cells where *cyclin D1* is upregulated in response to a gain of function for YAP/TEAD, in mouse neural progenitor cells [15]. Moreover, some of the functions of YAP are opposite to those of Yki. YAP, as a cofactor for p73 (a member of the p53 family of transcription factors) can promote apoptosis after DNA damage [26], [27], whereas Yki is clearly a suppressor of cell death in the fly eye. Finally, *Diap1* has been shown to be a direct target of Yki/Sd-mediated transcription [11], [12], but the same direct link is not yet established in mammals.

Mammalian homologs of the *Drosophila* Diap1 define a highly conserved family of intracellular proteins, the Inhibitor of Apoptosis Proteins (IAP) that suppress apoptosis induced by a variety of stimuli by binding specific intracellular proteases, primarily caspases 3, 7 and 9 [28], [29], [30]. In humans, eight family members have been identified (NAIP, c-IAP1, c-IAP2, XIAP, Survivin, Apollon, Livin and ILP2) [31], and only two in *Drosophila* (Diap1/2) [32].

Although the regulation of TEAD1 transcription is poorly understood so far, its expression is misregulated in several types of cancers. TEAD1 has been found either upregulated, for instance in prostatic or pancreatic cancers [33], [34], or conversely decreased in bladder or breast cancer, for example (as reported by the ONCOMINE database [35], [36], [37]). Nevertheless the functional outcome and significance of such TEAD1 modulations, as well as its *bona fide* target genes relevant to tumorigenesis remained elusive.

To gain insight into the role of TEAD1 in mammals, we explored the effect of modulating its expression level in HeLa cells and other human cell lines treated with the pro-apoptotic drugs, Staurosporine and Etoposide. Our molecular data demonstrate that both the downregulation and overexpression of TEAD1 increase the resistance of HeLa cells to induced apoptosis suggesting a proapoptotic function for TEAD1 and a non physiological cytoprotective effect for overexpressed TEAD1. We show evidence that overexpressed TEAD1 confers apoptotic resistance by titrating a cofactor required for its transcriptional activity. Our results further demonstrate that transcriptional up-regulation of the IAP family member Livin is required for TEAD1-associated cytoprotection. Using a repressive form of TEAD1 we show that Livin up-regulation induced by TEAD1 is indirect. Our data support a model in which TEAD1, together with a limiting cofactor, activate a repressor of Livin transcription. Interestingly, we show that overexpression of a mutated form of TEAD1 (Y421H) implicated in Sveinsson's chorioretinal atrophy that strongly reduces interaction of the mutant with YAP as well as its activation [38], [39] can induce Livin expression and protect cells from induced apoptosis, suggesting that YAP is not the cofactor involved in Livin repression. Taken together our data reveal a new, Livin-dependent, pro-apoptotic function for TEAD1 in mammals.

## Results

### Modulation of TEAD1 expression confers resistance to induced apoptosis

**Figure 1:**
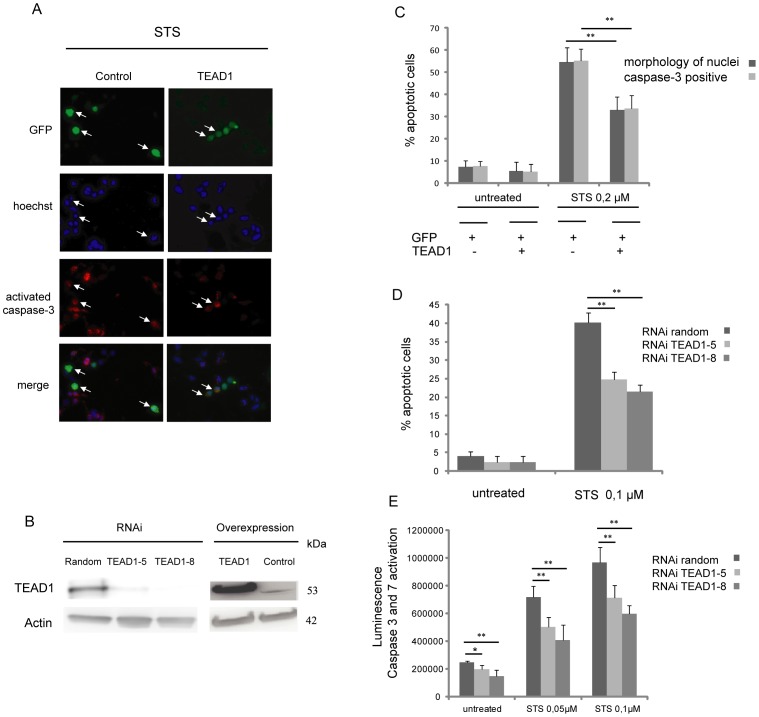
Modulation of TEAD1 expression in HeLa cells inhibits apoptosis induced by STS treatment. (A) 8.10^4^ Hela cells were transfected with 50 ng of GFP alone or together with 200 ng of TEAD1. After 24 h, the cells were treated with 0.2 µM STS for 3 h and stained with Hoechst (blue) and anti-Activated Caspase-3 antibodies (red). Apoptotic cells are indicated by arrows. (B) Western blot analysis of lysates from HeLa cells treated with siRNA (random, TEAD1-5 and TEAD1-8) or transfected with TEAD1 or a void plasmid (control). TEAD1 and Actin levels are revealed. (C) Percentage of apoptotic cells in response to TEAD1 transfection. HeLa cells were transfected as indicated with GFP and TEAD1 and were treated with STS (0.2 µM). Apoptotic cells were scored by nuclear fragmentation (Hoechst) or caspase-3 activation. Histograms indicate the percentage of apoptotic cells (mean +/− standard deviation) of at least four independent experiments. Asterisks indicate statistical significance, **P<0.01. (D) Percentages of apoptotic cells in the presence of random or TEAD1-specific siRNAs. 72 h after siRNA treatment, HeLa cells were treated with 0.1 µM STS, and apoptotic HeLa cells were detected by nuclear fragmentation (Hoechst). **P<0.01. (E). Luminescence-based detection of caspase-3 and caspase-7 activities in the presence of random or TEAD1-specific siRNAs. 72 h after siRNA treatment, the HeLa cells were treated for 3 h with 0.05 µM or 0.1 µM STS. Histograms display mean values from a minimum of three independent replicates. Error Bars indicate SD. Asterisks indicates statistical significance. The p values were calculated by Student's t test from six independent experiments. **P<0.01, *P<0.05.

Recent studies have revealed a possible role for TEAD proteins in apoptosis in mammals. Hence, overexpression of a transcriptionally active form of Tead2, (a chimeric TEA-containing the N-terminal region of Tead2 fused to the exogenous transactivation domain of VP16) in the mouse fibroblast NIH3T3 cell line, protected cells from apoptosis induced by Taxol [40]. *In vivo* studies showed that loss of function of TEAD1 (using a dominant negative containing the TEA domain and the surrounding sequences of TEAD1 only, but lacking the YAP-binding domain) leads to increased cell death in mouse neuronal progenitor cells [15]. Nevertheless, several of these results were not obtained using wild-type TEAD proteins, which for instance do not seem to affect proliferation when overexpressed [15], [16] and for which a putative effect on apoptosis had not yet been investigated. Importantly, increased apoptosis was observed in the ectoderm of *Tead1; Tead2* homozygous mutant mice [16] but the *bona fide* target genes relevant to cell survival had so far not been identified. We thus decided to explore the apoptotic role of the TEAD family of transcription factors, focusing on the effects of the modulation of TEAD1 expression on apoptosis in human HeLa cervical carcinoma cells. We first investigated the consequences of TEAD1 overexpression on induced apoptosis. Transfected cells were treated with Staurosporine (STS) a large spectrum inhibitor of protein kinases, that triggers the release of cytochrome *c* [41]. Apoptotic cells were scored by two complementary detection methods: 1) the nuclei morphology, as visualized by Hoechst staining, highlighting chromatin condensation and/or fragmentation; 2) the immunodetection of activated caspase-3 positive cells (Fig. 1A). The percentage of apoptotic cells revealed by the two methods was monitored in GFP-positive transfected cells. One day after transfection, Western blot analysis revealed increased levels of TEAD1 in transfected cells *versus* control cells transfected with a void plasmid (Fig. 1B). In untreated cells TEAD1 overexpression did not affect basal levels of cell death (Fig. 1C). Conversely, apoptosis induced by 0.2 µM STS was significantly decreased in TEAD1 overexpressing cells (Fig. 1C). The two detection methods yielded very consistent results, confirming that activated caspase-3 immunoreactivity as well as nuclear condensation and fragmentation are suitable apoptotic markers for these experiments. To gain insight into the mechanism of TEAD1-conferred apoptotic resistance, we also used two other types of cell lines displaying different biological features compared to HeLa cells (of tumoral origin with a p53 inactive phenotype). We overexpressed TEAD1 in BUA cells (a human fibroblast cell line) and in MCF7 (a p53 positive cell line derived from human mammary tumors). Since MCF7 cells do not express caspase-3 [42], for this set of experiments we monitored apoptosis solely by Hoechst staining. Consistent results were obtained after STS treatment, with a significant protection against induced apoptosis when TEAD1 was overexpressed (Fig. S1A). These results indicate that TEAD1-induced apoptosis resistance is p53-independent and not specific to tumor cells. Since expression of TEAD1 can protect cells from induced apoptosis, we conversely examined the effect of TEAD1 knock down on cell death. These experiments were performed on HeLa cells, since the level of TEAD1 mRNA in the two other cell lines (BUA and MCF7) is significantly lower (Fig. S1B). In addition, in HeLa cells, TEAD1 is the most prominently expressed TEAD family member (Fig. S1C), reducing the risk of a possible functional redundancy among TEAD paralogs that could compensate for the absence or reduction of TEAD1 [16]. Specific mRNA knockdown by two independent synthetic siRNAs (*TEAD1-5* and *TEAD1-8*) reduced TEAD1 to undetectable levels in Western blots (Fig. 1B). Treatment of cells with either of the TEAD1 siRNAs significantly protected HeLa cells from STS-induced cell death, as monitored by nuclear fragmentation (Fig. 1D). In agreement with this result, we observed that activation of the proapoptotic caspases-3 and 7 in response to STS (0.05 µM and 0.1 µM), and even basal levels of activation of these caspases, were significantly reduced upon TEAD1 knockdown (Fig. 1E). This latter result indicates that, in HeLa cells, TEAD1 has a pro-apoptotic function, since its loss of function renders cells more resistant to induced apoptosis. This is in contrast with previously published data in different cellular models [15], [16], indicating that the loss of function for TEAD1 sensitizes cells to apoptosis. The models used in these studies (normal tissue vs. cancer cells in our study) as well as the methodologies (dominant negative or knockout mice versus RNAi strategy in our study) can explain this difference. Moreover, apoptosis resistance observed in response to overexpressed wild-type TEAD1 is also novel, since a similar effect had previously been observed with a TEAD chimera fused with an exogenous transcriptional activation domain (Tead2-VP16), in another cell type (mouse fibroblasts) and using Taxol as inducer of apoptosis [40]. Taken together, our observations indicate that the alteration of wild-type TEAD1 expression levels is sufficient to promote a cytoprotective effect against pro-apoptotic stimuli. The fact that loss and gain of functions for TEAD1 have similar effects on cell survival suggests that TEAD1 overexpression mimics a loss of function phenotype by titrating a limiting cofactor required for its activity. This hypothesis is consistent with data previously observed in cultured cells [9].

### Induction of the two Livin isoforms by alteration of TEAD1 expression is required for apoptosis resistance

**Figure 2:**
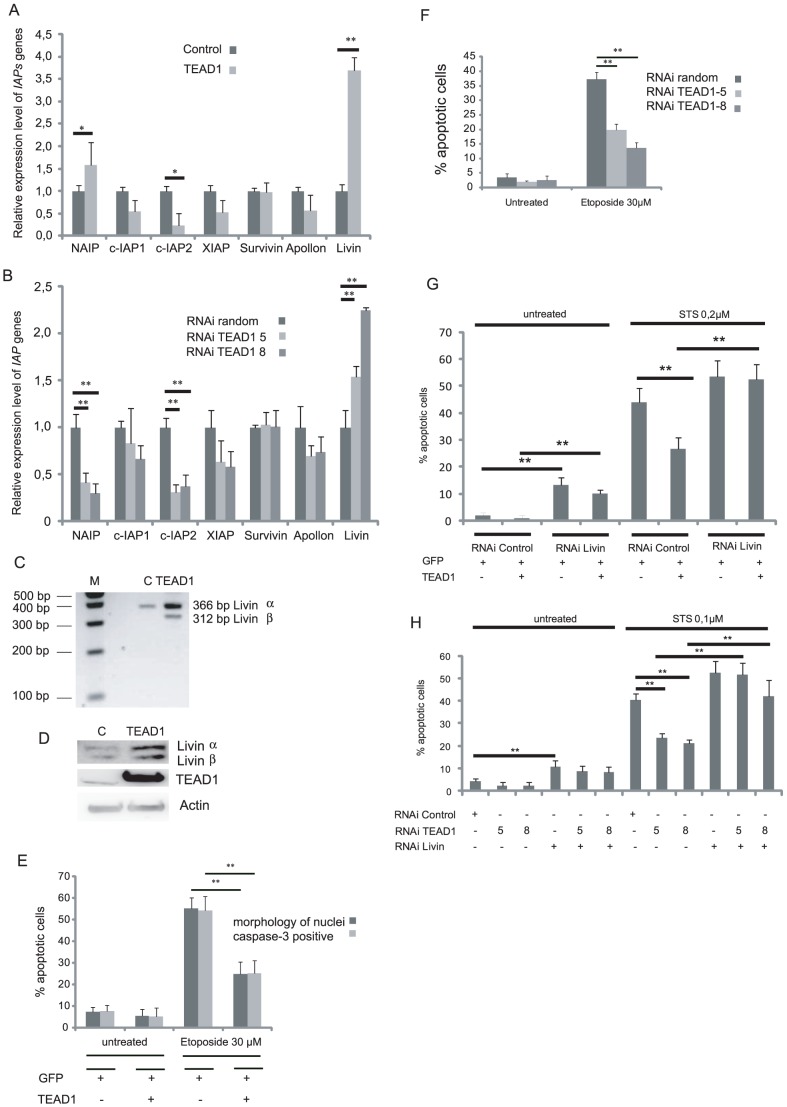
Induction of the two Livin isoforms by modulation of TEAD1 expression is required for apoptosis resistance. (A, B) RT-Q-PCR quantifications for the transcripts of seven *IAP* family members were performed on 10^5^ HeLa cells, either transfected with 400 ng of GFP-expressing and 800 ng of void or TEAD1-expressing plasmids (A) or treated with 10 nM of random or TEAD1-specific siRNA (B). *IAPs* mRNA quantification was normalized to endogenous GAPDH mRNAs for internal control. Values obtained for control transfection (A) or silencing (B) are normalized to 1 for each gene. Histograms display mean values from a minimum of three independent replicates. Error Bars indicate SD. Asterisks indicates statistical significance. The p values were calculated by Student's t test from six independent experiments. **P<0.01, *P<0.05. (C, D) HeLa cells transfected with 800 ng of void or TEAD1-expressing plasmids. Specific semi-quantitative RT-PCRs (C) and Western blot analyses (D) show induction of both α and β Livin isoforms in response to TEAD1. (E) Percentage of apoptotic cells in response to TEAD1 transfection. HeLa cells were transfected as indicated with GFP and TEAD1 and were treated for 18 h with Etoposide (30 µM). Apoptotic Hela cells were scored by nuclear fragmentation (Hoechst) or caspase-3 activation. Histograms indicate the percentage of apoptotic cells (mean +/− standard deviation) of at least four independent experiments. Asterisks indicate statistical significance, **P<0.01. (F) Percentages of apoptotic cells in the presence of random or TEAD1-specific siRNAs. 72 h after siRNA treatment, HeLa cells were treated with 30 µM Etoposide, and apoptotic HeLa cells were detected by nuclear fragmentation (Hoechst). Histograms indicate the percentage of apoptotic cells (mean +/− standard deviation) of at least four independent experiments. **P<0.01. (G) Percentage of apoptotic cells in the presence of random or Livin-specific siRNA. 48 h after siRNA treatment, 8×10^4^ HeLa cells were transfected with 50 ng of GFP alone or together with 200 ng of TEAD1-expressing plasmids. 24 h later, the cells were treated with 0.2 µM STS for 3 h and apoptotic cells were detected by nuclear fragmentation (Hoechst). **P<0.01. (H) Percentage of apoptotic cells in the presence of random, TEAD1 or Livin-specific siRNA. 72 h after siRNA treatment, the HeLa cells were treated with 0.1 µM STS for 3 h and apoptotic cells were detected by nuclear fragmentation (Hoechst). **P<0.01.

Previous studies in flies identified *Drosophila inhibitor of apoptosis 1* (*Diap1*) as a target of the TEAD-related transcription factor, involved in protection against apoptosis [11], [12]. In humans, the IAP family of *Diap1* homologs contains 8 members. We thus carried out RT-Q-PCRs to assess their expression levels (except for the pseudogene ILP-2) in TEAD1 gain- and loss of function experiments. The transfection of 10^6^ of cells with 800 ng of TEAD1 plasmid resulted in a 3.5 fold increase in the level of endogenous Livin mRNA whereas RNAs of other IAP family members were not significantly affected, except for c-IAP2 which was significantly reduced and for NAIP which was slightly induced (Fig. 2A). Similarly, the reduction of TEAD1 by either of the 2 independent siRNA resulted in 1.5 and 2.2 fold increases in the level of endogenous Livin mRNA whereas RNAs of other IAP family members were not significantly affected, except for c-IAP2 and for NAIP which were significantly reduced (Fig. 2B). Attention was focused on Livin up-regulation. To test whether this Livin up-regulation was specifically induced by TEAD1, other members of the TEAD family (TEAD2, 3 and 4) were overexpressed but failed to significantly increase Livin mRNA levels (Fig. S1D), indicating that Livin induction is TEAD1-specific. The DNA binding domain of TEAD proteins (TEA domain) is highly conserved [9], whereas the C-terminal domain that interacts with cofactors is more variable, and can bind different TEAD-specific interactors [43], [44]. The observed specificity for Livin up-regulation favors a model in which TEAD1 likely interacts with a specific cofactor involved in Livin regulation. *In vivo*, the two known spliced isoforms of Livin mRNA present different antiapoptotic properties [45], [46]. Using Jurkat cells as a model, Ashhab *et al.* (2001) showed that Livin αbut not βprotects cells from STS-induced apoptosis, whereas apoptosis induced by Etoposide treatments (a DNA topoisomerase II inhibitor inducing apoptosis through DNA damage) [47] was blocked only by the Livin β isoform. Since RT-Q-PCR cannot distinguish between these two splice variants, semi-quantitative PCR experiments with primers flanking the truncated region of exon 6 were performed in parallel. [45]. Our results indicate a TEAD1-dependent induction of both α and βLivin isoforms (Fig. 2C). We also ascertained that both protein variants accumulated in response to TEAD1 expression, demonstrating that mRNA up-regulation is coupled with increased protein production (Fig. 2D). Consistent with a possible role for Livin β up-regulation in TEAD1-mediated cytoprotection, we also observed that apoptosis induced by 30 µM Etoposide was significantly decreased in TEAD1 overexpressing cells (Fig. 2E). Similarly, the silencing of TEAD1 by two independent siRNAs also resulted in increased transcript levels for the two Livin isoforms (Fig. S1E), associated with a significant cytoprotection from apoptosis induced by 30 µM Etoposide (Fig. 2F). To gain further insight into the role of Livin up-regulation in TEAD1-mediated apoptosis protection, we performed Livin knockdown experiments in TEAD1 gain and loss of function contexts. siRNA-mediated targeting of its mRNA reduced Livin to undetectable levels for both mRNA isoforms (Fig. S1F). As expected, the basal apoptotic level was significantly increased upon Livin knockdown (Fig. 2G and 2H), consistent with previously published data [46], [48], [49], [50]. Importantly Livin RNAi also completely abolished the resistance to STS-induced apoptosis conferred by TEAD1 overexpression (Fig. 2G). A similar behaviour was observed in TEAD1 loss of function contexts. The cytoprotection from STS conferred by TEAD1 downregulation (*TEAD1-5* and *TEAD1-8* siRNAs) was also abolished upon Livin knockdown (Fig. 2H). Taken together our results demonstrate that Livin induction is necessary for TEAD1-dependend apoptosis protection.

### TEAD1overexpression indirectly up-regulates Livin expression

**Figure 3:**
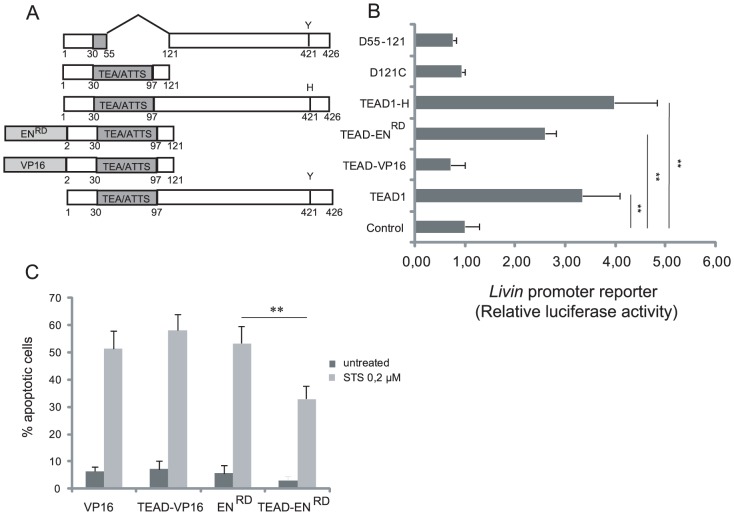
Structure-function analysis of TEAD1, using the *Livin* ML-IAPpro4 promoter readout. (A) Structural features of TEAD1 derived plasmids and structures of the TEAD-EN^RD^ and TEAD-VP16 plasmids. (B) Relative luciferase activity assessed after transfection of 8×10^4^ HeLa cells with the ML-IAPpro4 promoter luciferase reporter and 200 ng of the indicated TEAD1 derived plasmids. The data are expressed as the n-fold change relative to a negative control transfected with the ML-IAPpro4 promoter alone. Normalization of transfection efficiency was obtained by co-transfection of a β-galactosidase-expressing vector and assaying of β-gal activity in cell extracts. Error bars indicate standard deviation. **P<0.01. (C) Percentage of apoptotic cells upon TEAD1-VP16 or TEAD1-EN^RD^ transfection. HeLa cells were transfected with GFP and the indicated plasmids and were treated with 0.2 µM STS. Apoptotic cells were detected by nuclear fragmentation (Hoechst). **P<0.01, χ^2^.

To gain insight into the mechanism underlying TEAD1 dependent Livin up-regulation, HeLa cells were co-transfected with a luciferase reporter construct containing the upstream promoter of Livin (ML-IAP Pro4) [51] and with the TEAD1-expressing plasmid (Fig. 3A), which resulted in a 3.4-fold reporter activation, compared to the control (Fig. 3B). In agreement with the endogenous Livin induction experiments, this result strongly suggests that TEAD1 regulates the transcription of Livin rather than its mRNA stability. To assess which part of TEAD1 is necessary and/or sufficient for Livin expression, two deleted forms of TEAD1 were used, one containing the TEA domain only (Δ121C), and the other one containing a TEAD1 protein deleted of its TEA domain (Δ55-121) (Fig. 3A). As expected, the TEA domain did not affect the activity of a TEAD1 responsive reporter, while Δ55-121C could inhibit this responsive reporter, as previously published [1], [9] (Fig. S2A). Nevertheless, we did not observe any Livin induction in response to either truncated protein (Fig. 3B), indicating that a functional and entire TEAD1 protein is required for Livin induction.

To investigate whether TEAD1-induced apoptotic resistance and Livin induction are mediated through the transcriptional activity of TEAD1, a constitutively active form and a repressive form of TEAD1 were constructed by replacing its YAP binding domain [14] by the activation domain (AD) of VP16 (TEAD-VP16) or the repressor domain (RD) of Engrailed (TEAD-EN^RD^) (Fig. 3A). Indeed, previous studies in cultured cells showed that the activity of YAP AD is as potent as that of VP16 AD [52] and that a transcriptionally active form of TEAD1 recapitulates TEAD phenotypes when overexpressed [15], [40]. As expected, the TEAD-VP16 fusion protein stimulated a TEAD-responsive reporter construct in a dose-dependent manner (Fig. S2B) while the TEAD-EN^RD^ fusion protein strongly repressed that same reporter [53] (Fig. S2A).

**Figure 4:**
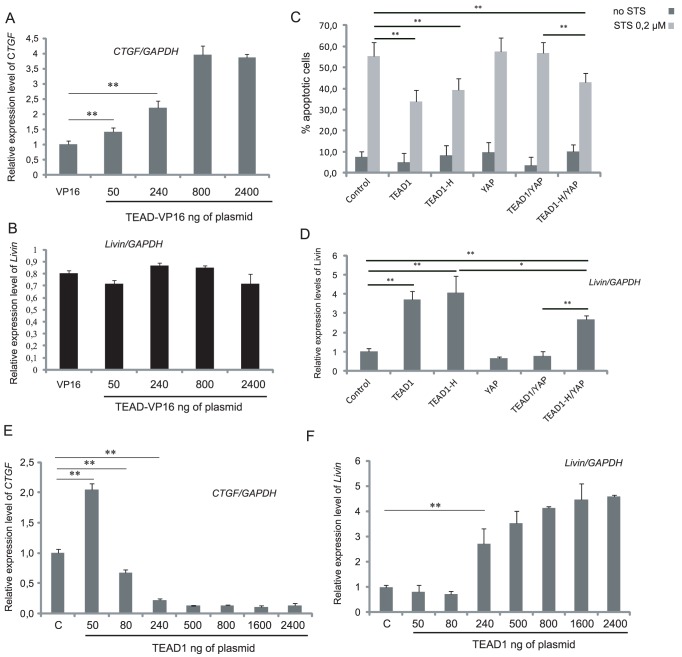
TEAD1-driven *Livin* up-regulation proceeds indirectly. (A, B) RT-Q-PCRs for *CTGF* (A) or *Livin* (B) mRNAs from HeLa cells transfeted with 400 ng GFP- and 800 ng VP16-expressing plasmid or increasing amounts of TEAD-VP16-expressing plasmid. mRNA quantification was normalized to endogenous *GAPDH* mRNAs for the internal control. Histograms show mean values for a minimum of three replicates. Bars indicate SD. (C) Percentage of apoptotic cells, as detected by nuclear fragmentation (Hoechst), upon treatment with 0.2 µm STS and transfection with void, TEAD1, TEAD1-H, or YAP plasmids alone or in combination. Histograms indicate the percentage of apoptotic cells (mean +/− standard deviation) of at least four independent experiments. *P<0.05, **P<0.01,. (D) RT-Q-PCR for *Livin* from HeLa cells transfected with TEAD1, TEAD1-H, or YAP plasmids alone or in combination. *P<0.05, **P<0.01, t-test. (E-F) RT-Q-PCR for *CTGF* (E) or *Livin* (F) mRNAs from HeLa cells transfected with increasing amounts of TEAD1. mRNA quantification was normalized to endogenous *GAPDH* mRNAs for internal control. Mean ± S.D. calculated from six independent replicates are displayed. *P<0.05, **P<0.01, t-test.

In contrast to TEAD1, TEAD-VP16 failed to induce the activity of the Livin reporter (ML-IAP Pro4) (Fig. 3B) and to protect cells from induced apoptosis (Fig. 3C). To confirm this result HeLa cells were then transfected with increasing concentrations of TEAD-VP16, and endogenous Livin expression was monitored by RT-Q-PCR. As a positive control, we quantified the mRNA levels of *CTGF*, a gene known to be directly activated by TEAD1 and YAP [13]. As expected, *CTGF* expression was induced by TEAD-VP16 in a dose-dependent manner (Fig. 4A). Conversely, TEAD-VP16 did not affect Livin mRNA expression (Fig. 4B), in accordance with results obtained with the Livin promoter. Nevertheless, on the contrary, the repressive form of TEAD1 (TEAD-EN^RD^) was able to activate the promoter of Livin (Fig. 3B) and to protect cells from induced apoptosis (Fig. 3C), similarly to wild-type TEAD1 (Fig. 1C). Since cytoprotection and Livin induction are not induced by the constitutively active TEAD-VP16, but on contrary by the repressor form TEAD-EN^RD^, our results indicate that TEAD1 does not upregulate Livin through direct binding of its promoter. Since our loss of function results indicate that endogeneous TEAD1 is also required to repress Livin expression in HeLa cells, taken together, our observations favor a model in which TEAD1 activates a repressor of Livin by interacting with a cofactor, required for TEAD1 transcriptional activity and thus induction of a Livin repressor. Most likely, the downregulation of this repressor, in response to TEAD-EN^RD^, or in response to cofactor titration by overexpressed wild-type TEAD1, would be responsible for the observed increase in Livin expression

To identify the putative cofactor involved in this activation, we further investigated the transcriptional co-activator YAP, one of the best-described cofactors for TEAD1 [13], [15]. YAP activity is regulated through its shuttling between the nucleus and the cytoplasm, under the control of the Hippo pathway. Indeed, activation of the Hippo pathway leads to YAP phosphorylation and its exclusion from the nucleus. Besides its partnership with TEAD1, YAP also interacts with and increases the activity of p73, a member of the p53 family that positively regulates pro-apoptotic genes [26], [54]. Thus, it is conceivable that the YAP/TEAD1 interaction competes with the YAP/p73 interaction resulting in a decrease in p73 pro-apoptotic activity.

To investigate whether the YAP/TEAD1 interaction is involved in anti-apoptotic properties of TEAD1 expression, two strategies were used. In one approach, TEAD1 was overexpressed with YAP. When YAP alone was overexpressed, no effect on the susceptibility to apoptosis or on Livin induction was observed (Fig. 4C,D). Interestingly, when TEAD1 and YAP were co-expressed, the anti-apoptotic effect of TEAD1, as well as Livin induction, were completely abolished (Fig. 4C,D), whereas the expression of the *CTGF* target gene of TEAD1/YAP was highly induced (Fig. S1G). These results indicate that YAP overexpression does not modulate the susceptibility of HeLa cells to apoptosis. However experiments in which TEAD1 and YAP were overexpressed indicate that YAP can interfere with the anti-apoptotic activity of TEAD1 possibly through direct interaction between YAP and TEAD1.

To examine this possibility, we analyzed in parallel how a mutant TEAD1 protein unable to interact with YAP affects the susceptibility to apoptosis or Livin induction. Advantage was taken of the mutation of a highly conserved tyrosine in the YAP-binding domain of TEAD1, (TEAD1-Y421H, hereafter referred to as TEAD1-H) (Fig. 3A). This mutation causes the human genetic disease known as Sveinsson's chorioretinal atrophy [38], and strongly reduces both YAP/TEAD1 interaction and activity [55]. Contrary to wild-type TEAD1, TEAD1-H was unable to activate the expression of the TEAD1 responsive reporter when transfected with YAP, confirming that the interaction with YAP is severely impaired (Fig. S2A). HeLa cells transfected with wild-type *versus* mutant forms of TEAD1, showed no difference in their resistance to induced apoptosis or Livin expression levels (Fig. 4C,D). To further confirm this result, we analyzed whether TEAD1-H was able to activate the Livin promoter reporter ML-IAPpro4. As shown in Fig. 3B, similarly to TEAD1, TEAD1-H increases Livin promoter activity. However, contrary to what was observed with wild-type TEAD1, YAP overexpression did not abolish the antiapoptotic effect of TEAD1-H (Fig. 4C), even if Livin up-regulation was partially decreased (Fig. 4D), potentially due to some residual TEAD1-H/YAP interaction. Altogether, the results obtained with TEAD1-H, which has a severely reduced YAP-binding ability, clearly indicate that the TEAD1 anti-apoptotic properties as well as Livin regulation are YAP independent and do not favour the hypothesis that TEAD1 acts by competing with the p73/YAP complexes. On the other hand, our observations showing that overexpression of YAP prevents the anti-apoptotic effects of TEAD1 indicate that YAP can interfere with the anti-apoptotic properties of TEAD1. This can be explained by assuming that YAP overexpression impairs TEAD1-mediated cytoprotection by competing with another cofactor for binding to the overexpressed-TEAD1. TEAD1-YAP dimers would thus prevent overexpressed TEAD1 from binding and titrating the cofactor required to activate a Livin repressor. Accordingly, TEAD1/YAP expression leaves Livin expression level low, comparable to that observed in the control (Fig. 4D), and does not protect from apoptosis (Fig. 4C).

Yet, even if it is well established that TEAD1 must interact with cofactors to activate transcription [7], [8], TEAD1 overexpression in cultured cells may inhibit their transcriptional functions by “titrating” co-activator proteins [9]. The existence of such highly limiting, possibly cell-specific, titratable transcriptional co-factor(s) has been deduced from transfection analyses where activation of a cognate reporter is severely reduced upon TEAD1 overexpression [1], [9], [56], [57], [58] (Fig. S2A). If TEAD1 represses Livin (as suggested by the TEAD1 RNAi and TEAD-EN^RD^ transfection experiments) by interacting with a limiting cofactor required for the activation of a Livin repressor, higher doses might titrate this cofactor and allow subsequent Livin expression. To gain further insight into the mechanism by which modulation of TEAD1 expression positively regulates Livin expression, 10^6^ cells were treated with increasing amounts of TEAD1, ranging from 50 ng to 2400 ng of plasmid. As positive control, the mRNA of the TEAD1-YAP target gene *CTGF* [13] was quantified. As expected, after a slight induction, due to the presence of endogenous YAP, expressed to levels 20 folds higher than TEAD1 in HeLa cells (data not shown), a dose dependent repression of CTGF expression was observed (Fig. 4E). Conversely, a dose-dependent induction of Livin mRNA expression, starting at 240 ng and reaching a plateau after 800 ng of transfected plasmid was detected (Fig. 4F). This latter observation together with the RNAi and TEAD-EN^RD^ results, strongly suggests that TEAD1 overexpression does not directly regulate Livin transcription, but merely titrates a cofactor necessary for the activation of a Livin repressor.

## Discussion

Several lines of evidence indicate a physiological role for TEAD1 in mammalian developmental processes. For example, TEAD1 plays a vital role in fetal heart development, and post-natal heart function. TEAD1 is constitutively expressed in cardiac and skeletal muscles in pigs, mice and humans [56], [57] where it regulates the expression of many skeletal muscle-specific genes that contain the M-CAT motif (TEAD1 protein binding site) [58], [59]. Its disruption in mice leads to heart defects and embryonic lethality between embryonic days 11 and 12 [60] while its overexpression in the mouse heart can induce age-dependent dysfunction [61]. Moreover, recent data support an anti-apoptotic role for TEAD proteins during mouse development, based on *Tead1* and/or *Tead2* loss of function experiments [15], [16]. However, the effect of a gain of function for wild-type TEAD proteins had not yet been explored either in these models or in cancer cell systems, since only a transcriptionally active form of TEAD was investigated in previous studies [40]. Here we show that overexpression or loss of function for the wild-type TEAD1 protein is sufficient to protect HeLa cells from induced apoptosis and can induce a significant up-regulation of both isoforms of the Livin protein that belongs to the IAP family. The anti-apoptotic features of Livin are well known [45], [65], [66]. Our Livin RNAi experiments not only confirm these previously published data, but also demonstrate that Livin up-regulation is required for TEAD1-induced apoptotic protection. We investigated the mechanisms underlying both apoptosis protection and Livin induction. The results obtained led us to conclude that Livin up-regulation in response to TEAD1 overexpression is indirect. Indeed, TEAD-VP16 activates a TEAD1 responsive reporter construct, as well as the TEAD/YAP direct target *CTGF*, but fails to increase the activity of the Livin promoter and apoptosis resistance. By contrast, TEAD-EN^RD^, that can repress a TEAD1 responsive reporter construct, activates the Livin promoter and confers apoptotic resistance. This excludes the possibility that TEAD1 binds to Livin regulatory sequences to activate its expression but favors a model in which wild-type TEAD1 activates a repressor of Livin by interacting with a cofactor. In conditions in which we express TEAD-EN^RD^, this repressor is down regulated, increasing Livin expression. This model is in good agreement with TEAD1 knock down (Fig. 2B) and overexpression (Fig. 4F) results. The latter experiment also suggests that TEAD1 needs to interact with a limiting cofactor to activate the expression of a Livin repressor. The fact that the Livin promoter activity or its mRNA levels are not further reduced in response to TEAD-VP16, suggests that the Livin repressor is endogenously expressed at levels high enough to achieve maximal Livin repression. This is in agreement with the low endogenous levels of Livin observed in HeLa cells compared to other cell lines, as melanoma cells (data not shown). Our data exclude the possibility that the TEAD1 cofactor is YAP. Indeed, TEAD1-H, which has strongly impaired YAP-binding ability, stimulates the activity of the Livin promoter and confers apoptosis resistance as efficiently as a wild-type TEAD1 protein. Furthermore, we showed that other members of the TEAD family (TEAD2, 3 and 4) fail to significantly increase Livin mRNA levels (Fig. S1D) while they are able to interact with YAP [14]. By using deleted forms of TEAD1, we showed that both the N-terminus and C-terminus domains of TEAD1 are required for Livin up-regulation. This indicates that the interaction between TEAD1 and a cofactor different from YAP likely involves spatial relationships between specific amino acid residues located in the N- and C-terminus domains of TEAD1. A similar spatially constrained interaction seems to occur between TEAD1 and other cofactors such as MEF2. Indeed, TEAD1 and MEF2 physically interact through the TEA and MAD box domains but additional sequences in the activation domains of both proteins are required for *in vivo* association [59]. Our results (Fig. 4C,D) and published data [62], [63] suggest that, in HeLa cells, YAP overexpression or the Hippo signaling pathway does not play a role in apoptosis regulation. However, we observed that YAP up-regulation can modulate the anti-apoptotic effect of overexpressed TEAD1. Increased YAP levels have been reported in a large variety of cancers, and might interfere with the role of TEAD1 in apoptosis. Although its transcriptional regulation is poorly understood, TEAD1 is up-regulated in several types of cancers such as prostatic or pancreatic cancers [33], [34], reaching up to 300 fold induction in Kaposi's sarcoma, (Gene Expression Atlas : www.ebi.ac.uk/gxa). However, analysis of the Oncomine database (www.oncomine.org) also reveals that TEAD1 is downregulated in other types of cancers, such as bladder, renal or breast cancers. In agreement with our results, it has been shown that in cancers where TEAD1 is overexpressed, such as in prostate cancers [33], Livin is also up-regulated [64], [65]. However, interestingly, a modest but significant, increase in Livin is also observed in other types of cancers where TEAD1 is down-regulated, such as breast, renal or bladder cancers (www.oncomine.org). The data presented in our study, in which a similar apoptotic resistance and Livin up-regulation is observed upon TEAD1 knockdown or overexpression, provide a conceptual framework to reconcile such discrepancies observed in different cancer types.

Furthermore, TEAD1 directly regulates the transcription of Mesothelin (MSLN) that is highly expressed in several cancers and is a good candidate for a diagnostic marker [34]. Interestingly, none of the expression patterns of the known TEAD1 cofactors such as YAP matched the expression of MSLN in pancreatic cancers or in other cell line models used here. This strongly suggests the existence of cofactors different from YAP, required to mediate TEAD1 activity in malignancies. Interesting potential candidates include the Vestigial-like proteins (Vgll1-4), the mammalians homologues of the *Drosophila* transcriptional coactivator Vestigial (Vg). Although Vgll1 and YAP are not homologous, a recent study has shown that Vgll1 interacts with TEAD by sharing two out of the three interfaces required for the interaction with YAP [66]. In addition, a competition was observed between Vgll1 and YAP for their binding to TEAD. However, the authors have shown that some of genes regulated by YAP/TEAD, such as IGFBP-3, SERPINE1 or FGF1, are not up-regulated by Vgll1. Conversely the Insulin-like growth factor binding protein-5 (IGFBP-5) is up-regulated by Vgll-1 but not by YAP. This clearly indicates that TEAD factors can interact with different cofactors to regulate specific sets of target genes. To further address the role of TEAD1 in cytoprotection, in physiological or pathological (cancers) conditions, additional studies will be required to determine the cofactor(s) with which TEAD1 must specifically interact to regulate the expression of the Livin repressor.

## Materials and Methods

### RNA extraction, reverse transcription (RT) and Quantitative PCR (Q-PCR)

For PCR analysis, 10^5^ transfected, GFP positive cells were sorted by FACS analysis and collected into RNAse-free tubes. Total RNA extraction, cDNA synthesis and Q-PCR were performed as described previously [67]. Amplifications of the *GAPDH* and *RPL13* genes were performed to ensure unambiguous comparisons between cDNAs from different samples. Primers and annealing temperatures for all genes are indicated in Table S1. For each gene, the values were averaged over at least three independent measurements. Three independent RNA isolations were performed for all experiments, and the means were calculated. For qualitative RT-PCR of Livin, primers and experimental conditions were according to [45].

### Cells, plasmids, transfections and apoptosis induction

HeLa and MCF7 (ATCC) as well as BUA cells, and human fibroblasts transformed with the sarcoma virus SV-40, (a gift of C. Alcaide) [69] were maintained in RPMI 1640 medium with 5% foetal calf serum for HeLa and 10% for MCF7 and BUA cells, 100 U/ml penicillin, and 100 µg/ml streptomycin at 37°C in 5% CO_2_. 8×10^4^ cells were plated onto 12-well plates the day before transfection. The TEAD1, TEAD1 Δ55-121, (gifts from I. Davidson) [1], TEAD1-Y421H, and pEGFP (GFP) (Clontech) cDNAs were subcloned into the pXJ40 vector; the YAP1, MST2 and LAST1 cDNAs (gifts from X. Yang) were subcloned into the pCDNA3.1 vector to allow expression of these cDNAs under the control of a CMV promoter. TEAD-VP16 and TEAD-EN^DR^ were obtained by cloning the TEA domain (amino acids 15–121) of TEAD1 into the plasmids pCS2-VP16 and pCS2-EN^DR^ (gift of P. Thiebaud) [68]. HeLa, BUA and MCF7 cells were transfected with these plasmids (individually or in combination), together with GFP, using the Effectene Transfection Reagent (Qiagen), according to the manufacturer's instructions. To induce apoptosis, the cells were exposed 24 h postransfection to different concentrations of Staurosporine (STS) (Sigma), for 3 h, and to Etoposide (Sigma) for 18 h. The ML-IAPpro4 plasmid (a gift of D. Vucic) contains a functional 1.6 kb region of the promoter of Livin cloned into the pGL4.10 plasmid [51]. The −2570/−2518-TK164-luc plasmid (a gift of A. Payne) contains a 53 bp fragment from the promoter of the 3β-hydroxysteroid dehydrogenase-isomerase (3βHSD) promoter cloned upstream of a thymidine kinase minimal promoter. This 53 bp region of the 3βHSD promoter contains binding sites for the TEAD factor. For the luciferase assay, 8×10^4^ cells were transfected with 200 ng of the ML-IAPpro4 promoter luciferase reporter plasmid or with 200 ng of the −2570/−2518-TK164-luc plasmid, alone or in combination with the plasmids described above. 48 h post-transfection, firefly luciferase activities were assayed with the Promega luciferase assay kit. All transfections were normalized by cotransfection of a β-galactosidase expression vector. The β-galactosidase activity was evaluated with ONPG assays. All experiments were performed at least six times.

### Apoptosis assays and statistical analysis

Nuclear morphology was visualized using Hoechst 33342 (Sigma) staining for 20 min at room temperature. Apoptotic cells were scored when the nuclei displayed chromatin condensation and/or fragmentation. Caspase-3 positive cells were visualized by antibodies specific for activated caspase-3 protein (clone C92-605, BD Pharmigen) and scored to detect apoptotic cells. The ratio of apoptotic to viable cells revealed by the two methods was determined by fluorescence microscopy and 400 cells were scored for each sample. A measure of the activities of casapse-3 and caspase-7 was obtained using a luminogenic substrate (Caspase-Glo 3/7 Assay, Promega), according to the manufacturer's instructions. Experiments were repeated at least four times. To estimate the significance of the differences between means the parametric statistical Student's t test was used.

### Western Blot Analyses

HeLa cells transfected as described above were harvested 24 h post-transfection washed with PBS, and lysed with RIPA buffer (50 mM Tris-HCl pH 7.4, 150 mM NaCl, 1% NP40, 0.25% Na-deoxycholate, 1 mM PMSF, and 1× Roche complete mini-protease inhibitor cocktail). NuPAGE® Novex® 4–12% Bis-Tris Gels were loaded with 40 µg of total proteins and transferred to PVDF membranes. Antibodies against the following proteins were used: TEAD1 (polyclonal Santa Cruz Biotech sc-23793 (1∶50)); Livin (monoclonal Imgenex clone 88c570 (2 µg/ml)) and Actin (polyclonal Santa Cruz Biotech H-196 (1∶1000)). The immunological reaction was detected by means of a peroxidase-linked secondary antibodies and immunoreactive bands were visualized using the Supersignal West Pico Chemioluminescent Substrate Kit (Pierce).

*siRNA-* siRNA against *TEAD1 (TEAD1-5* 5′-CGATUUGUAUACCGAAUAA and *TEAD1-8* 5′-GAAAGGUGGCUUAAAGGAA *), Livin* or negative-control RNA were chemically syntesized (Dharmacon Research, Lafayette, USA). Synthetic siRNAs were transfected with TransIT-TKO Transfection Reagent (Euromedex) according to the manufacturer's instructions.

## Supporting Information

Figure S1 (A) Percentage of transfected apoptotic cells in response to TEAD1 overexpression. MCF7 and BUA cells were transfected with GFP and a void or TEAD1-expressing plasmid, and were treated with 0.1 µM STS. Apoptotic cells were identified by nuclear fragmentation (Hoechst). Histograms indicate the percentage of apoptotic cells (mean +/− standard deviation) of at least four independent experiments. Asterisks indicate statistically significance, **P<0.01. (B) Relative amount of *TEAD1* mRNA in the three cell lines HeLa, MCF7 and BUA, quantified by RT-Q-PCR. *GAPDH* and *RPL13* (Ribosomal Protein L13) were used as internal controls to normalize the *TEAD1* mRNA levels. (C) Relative mRNA levels for the four TEAD genes in HeLa cells. GAPDH served as internal control to normalize the *TEAD1*, *2*, *3* and *4* mRNAs. *TEAD1* levels are set to 1. *P<0.05, **P<0.01, t-test. (D) Detection of Livin expression by RT-Q-PCR after overexpression of the indicated TEAD-expressing vectors. (E) Specific semi-quantitative RT-PCR for Livin α and β mRNA in HeLa cells treated with a random or TEAD1-specific siRNAs. β-actin was used as loading control. (F) Specific semi-quantitative PCR for Livin α and β mRNA in HeLa cells treated with a random or Livin-specific siRNA. β-actin was used as loading control. (G) Detection of *Livin* and *CTGF* expression by RT-Q-PCR after overexpression of the indicated expressing vectors.
(EPS)

Figure S2 (A–B) Relative luciferase activity in 2×10^5^ HeLa cell transfected with 200 ng of −2570/−2518-TK164-luc plasmid (TEAD1 responsive luciferase reporter) and with 200 ng of TEAD1, TEAD-EN^RD^, YAP, TEAD1-Y, Δ55-121 and Δ121C plasmids (A), or 200 ng of TEAD1, YAP or TEAD-VP16 plasmids (200 and 400 ng) (B). The data are expressed as the n-fold change relative to control cells transfected with −2570/−2518-TK164-luc plasmid alone. Normalization of transfection efficiency was performed by co-transfection of a β-galactosidase expression vector followed by β-gal activity assays in cell extracts. Error bars indicate standard deviation, *P<0.05, **P<0.01.
(EPS)

Table S1 Primers and annealing temperatures for all the genes used in this study.
(XLSX)
